# Editorial: Community series - reducing the burden of age-related disease in relation to osteoporosis, sarcopenia and osteosarcopenia, volume II

**DOI:** 10.3389/fmed.2023.1344694

**Published:** 2023-12-18

**Authors:** Ozra Tabatabaei-Malazy, Patricia Khashayar, Arshed Ali Quyyumi, Iraj Nabipour, Mohammad Hossein Dabbaghmanesh, Leith Zakraoui, Bagher Larijani

**Affiliations:** ^1^Non-Communicable Diseases Research Center, Endocrinology and Metabolism Population Sciences Institute, Tehran University of Medical Sciences, Tehran, Iran; ^2^Osteoporosis Research Center, Endocrinology and Metabolism Clinical Sciences Institute, Tehran University of Medical Sciences, Tehran, Iran; ^3^Center for Microsystems Technology, Imec & Ghent University, Zwijnaarde-Gent, Belgium; ^4^Emory Clinical Cardiovascular Research Institute, Emory University, Atlanta, GA, United States; ^5^The Persian Gulf Marine Biotechnology Research Center, The Persian Gulf Biomedical Sciences Research Institute, Bushehr University of Medical Sciences, Bushehr, Iran; ^6^Endocrinology and Metabolism Research Center, Shiraz University of Medical Sciences, Shiraz, Iran; ^7^Department of Rheumatology, Mongi Slim Hospital, La Marsa, Tunis, Tunisia; ^8^Endocrinology and Metabolism Research Center, Endocrinology and Metabolism Clinical Sciences Institute, Tehran University of Medical Sciences, Tehran, Iran

**Keywords:** osteoporosis, sarcopenia, osteosarcopenia, aging, frailty, risk factors, bone mineral density, resistance exercise

Considering the accelerated aging rate, doubled to 1.5 billion by 2050, the cost and burden of related health problems are increasing. One of the more prevalent health problems among the elderly population is frailty that finally can result in sarcopenia and osteoporosis which may increase the fracture rate. This issue therefore aimed to report novel advancements in these two areas, focusing on new preventive measures and treatment options. The effect of certain co-morbidities on these two conditions were also highlighted, suggesting that preventing osteoporosis/sarcopenia can also reduce other co-morbidities among the elderly, improving their quality of life.

Three of the articles published in this issue were focused on sarcopenia (Merchant et al., Diniz de Salles et al., Hou et al.). The beneficial effects of nutrition in the pre-frailty phase on the prevention or reduction of frailty and disability risk in older adults is well known (1). Leucine, and leucine-enriched protein supplements in the range of the recommended dietary allowance (RDA) for protein in older adults (0.8 g/kg per day), together with resistance exercise, have been shown to be beneficial for muscle mass, physical function, and systemic inflammation. However, the effect of an additional dose of protein on RDA when combined with exercise in the elderly population is not yet known. In a non-randomized trial study, Merchant et al. assessed the short-term effect (3 months) of a diet enriched with an additional protein and leucine supplementation with/without exercise on the physical function, muscle mass, and systemic inflammation in prefrail older adults who had received RDA protein. They found a significant improvement in body cell mass, and systemic inflammation in both groups; short physical performance battery (SPPB) test, gait speed, 5× sit-to-stand (STS), and muscle mass, however, improved significantly only in the nutrition+ exercise group. Since these effects were not sustained after a 3-month follow-up, their findings should be confirmed in future randomized trials with a larger number of at-risk elderly.

Elderly patients are at increased risk of postoperative complications (2), which require rapid recognition and treatment. Otherwise, they can lead to a cascade of events that may result in loss of independence, some degrees of disability, worsening of quality of life, higher treatment-related costs, and higher mortality. Diniz de Salles et al. aimed to evaluate the association between sarcopenia and frailty in an elderly population admitted to hospital for non-emergency surgical procedures. This is while the association between sarcopenia and frailty is still unclear, while several studies have shown the influence of sarcopenia on frailty over time (1, 3). The secondary objective of the study was to evaluate the correlation of sarcopenia and frailty with postsurgical outcomes. This is mainly because sarcopenia and frailty are believed to have significant adverse effects on the postoperative outcomes (4). In this observational study, frailty was assessed using the modified frailty index (MFI-11). Sarcopenia, on the other hand, was measured through (a) thickness and echogenicity on ultrasound; (b) handgrip strength on dynamometry; and (c) gait speed. They found sarcopenia, in all its domains, was associated with frailty. Unfavorable surgical outcomes were also associated with these two conditions. Diniz de Salles et al. also suggested that screening for sarcopenia and frailty in the elderly patients undergoing elective surgery is relevant, easy to perform, and helps with perioperative risk reduction in this population.

Sarcopenia can be defined by various signs and symptoms, one of which is low muscle strength. There are several tools and methods to diagnose the mass and strength of muscle in sarcopenic people (5) such as handgrip strength test, and dual-energy X-ray absorptiometry (DEXA). Hou et al. in a cohort study with a 600-day follow-up determined the impact of the uremic toxins on the frequency of low handgrip strength in 75 participants divided into three groups: control, chronic kidney disease (CKD), and end-stage renal disease (ESRD). Although, they found a similar rate of sarcopenia between groups, handgrip strength and serum level of indoxyl sulfate, a protein-bound uremic toxin were lower and higher accordingly in ESRD patients. Indoxyl sulfate can impair the function of mitochondria of skeletal muscle cells and muscle anabolism by inducing oxidative stress (6). Moreover, the hospitalization rate was higher in patients with ESRD. They concluded the low handgrip strength to be predictive of hospitalization.

The other articles were focused mainly on osteoporosis. Osteoporosis is a systemic skeletal disease characterized by loss of bone mass and micro architectural integrity that leads to increased bone fragility and risk of fracture (7). With the advancements in science and technology, the quality of life and health status have significantly improved in the past decades. There are still huge differences in the living style, socioeconomic conditions, and medical status, such as smoking, education level, economic disparity, and chronic diseases, in various regions of China (8, 9). Such differences may have contributed to the disparities in the prevalence of osteoporosis in these regions. To address this issue, Wang J. et al. assessed the prevalence of osteoporosis and osteopenia as well as the associated risk factors in the China Community- based Cohort of Osteoporosis (CCCO). The multicenter cross-sectional study was conducted on 19,848 middle-aged and elderly permanent residents of seven representative Chinese regions (10, 11). The bone mineral density at the lumbar vertebrae and hip was determined using the dual-energy X-ray absorptiometry densitometer. Serum levels of bone metabolism markers were also measured. This study revealed dramatic regional differences in the prevalence of osteoporosis in China, with females, those aged 60 or older, with low BMI, low education level, current regular smokers, and with a history of fracture being at a higher risk of osteoporosis. They suggested that more preventive measures and treatment options should be focused on populations with such risk factors.

The maintenance of bone mass is negatively affected by metabolic dysfunctions such as chronic hyperglycemia (12). As a result, osteoporosis is more prevalent among patients with diabetes mellitus. In addition, it is well known that lipid profile disturbances especially high-density lipoprotein cholesterol (HDL-C) and apolipoprotein A1 (APOA1), its major protein component, have key roles in bone mass maintenance through affecting osteoblasts differentiation (13). However, there are controversies regarding their association with bone mineral density (BMD) values. Wang W. et al. investigated Chinese postmenopausal diabetic women for such associations. They found a remarkable association between APOA1 and osteocalcin level (which inhibits bone formation), lumbar BMD, and osteoporosis in contrast to HDL-C.

Cardiovascular disease and osteoporosis are common diseases in older adults, and both are associated with high morbidities (14). Yang and Huang study performed a multivariate logistic regression and stratified analysis to explore the possible relationship between BMD and the risk of CVD in older adults aged over 60 years. This study is expected to provide more guidance on early monitoring and clinical prevention. The cross-sectional study of 2,097 people aged over 60 years speculated that DXA examination or targeted prevention strategies, such as increased sun exposure, appropriate physical exercise, and calcium or vitamin D supplements, should be considered for CVD patients. Meanwhile, for osteoporotic patients or those at high risk of fracture, active anti-osteoporosis drug therapy can increase BMD, improve bone quality, and reduce cardiovascular complications to a certain extent. Negative non-linear relationship was noted between the femur BMD levels and the prevalence of CVD in people aged over 60 years with an inflection point of 0.741 gm/cm^2^. No significant differences were found between age, gender, and the comorbidities subgroups. Bone loss therefore was considered as a new risk factor for CVD. This is while future studies are needed to make a comprehensive assessment of combined dietary and biochemical indicators. This also points out the importance of preventive measures for osteoporosis to indirectly reduce the prevalence of CVD, the world's biggest killer of humans.

Current osteoporosis medications have drawbacks such as possible side effects and having slow onset, therefore developing osteoporosis drugs with faster onset and fewer side effects is essential. Therefore, the Shih et al. study investigated the effects of topical SDTL-E (15) applied for 20 days in ovariectomized (OVX)-induced osteoporosis rat models. The changes in estradiol, various bone turnover markers such as serum alkaline phosphatase (ALP) activity (16), serum and urinary calcium (17), bone mineral density (BMD), various bone mechanics indicators and bone histology were assessed to understand the mechanism of action and the therapeutic efficacy of SDTL-E on osteoporosis. The results demonstrated that the 20-day treatment with topical SDTL-E can improve bone strength and trabecular bone structure in OVX-induced osteoporosis rats. The underlying mechanisms include restoring estradiol levels as well as reducing bone turnover, net bone resorption, bone calcium loss, and calcium excretion through the kidneys. These findings suggest topical application of the plant extract is an efficient potential new approach for rapid treatment of osteoporosis.

One of the most common osteoporotic complications is osteoporotic vertebral fracture (OVF). OVF can lead to loss of height, acute and chronic pain, decreased quality of life, and increased fracture risk (18). Since, falls are known as the main risk factor for fracture in patients with OVF, improving body balance by exercise is believed to have an important role in reducing the future fracture risk (19). Li et al. conducted a systematic review and meta-analysis to assess the effects of resistance and balance exercises in patients with OVF. They found exercising improved quality of life, visual analog pain scale, Timed Up and Go, falls efficacy scale international, kyphosis, and functional reach. These beneficial effects were considerable when training continued for at least 10 weeks.

Odontoid fractures are another relatively common fracture of the spine (C2) vertebral body among the elderly. Despite its prevalence, there are controversies in its treatment. Several studies have suggested surgical management, suggesting it can result in more biochemical stability and fusion compared to the conventional therapies (20). This is while the surgical risks are high among the elderly population. As a result, maintaining the right balance between the risks and benefits of the surgery is challenging. Lenga et al. in a retrospective study with a 5-year follow-up assessed the morbidity and mortality rate of peri- and post-surgery of C1/C2 posterior screw fixation technique against the associated risk factors and mortality. Although they found a high rate of morbidity and mortality in octogenarians, they recommended spine surgery to achieve bone union and preserve of neurological status.

To conclude, all articles of this Research Topic sum up the current information and highlight the current research gaps and elucidate the path for future research on the topic. The findings of the published studies in the current Research Topic have been proposed some acceptable modalities and strategies for management of Osteoporosis, Sarcopenia and Osteosarcopenia, and also improvement of the patients' quality of life.

## Author contributions

OT-M: Validation, Writing—original draft, Writing—review & editing. PK: Validation, Writing—original draft, Writing—review & editing. AQ: Validation, Writing—review & editing. IN: Validation, Writing—review & editing. MHD: Validation, Writing—review & editing. LZ: Validation, Writing—review & editing. BL: Supervision, Validation, Writing—review & editing.
